# Conversion surgery after response to zolbetuximab-based chemotherapy in Claudin 18.2-positive gastric cancer: a case report

**DOI:** 10.1093/jscr/rjag495

**Published:** 2026-06-23

**Authors:** Shinya Ohno, Tomonari Suetsugu, Hironobu Takeuchi, Sho Miyazaki, Tatsuki Kawahara, Kazuo Yamamoto, Shunya Kiriyama, Kakeru Tawada, Yoshinori Iwata, Shuji Komori, Chihiro Tanaka, Narutoshi Nagao, Masahiko Kawai

**Affiliations:** Department of Surgery, Gifu Prefectural General Medical Center, 4-6-1 Noisshiki, Gifu-city, Gifu-prefecture 500-8717, Japan; Department of Surgery, Gifu Prefectural General Medical Center, 4-6-1 Noisshiki, Gifu-city, Gifu-prefecture 500-8717, Japan; Department of Surgery, Gifu Prefectural General Medical Center, 4-6-1 Noisshiki, Gifu-city, Gifu-prefecture 500-8717, Japan; Department of Surgery, Gifu Prefectural General Medical Center, 4-6-1 Noisshiki, Gifu-city, Gifu-prefecture 500-8717, Japan; Department of Surgery, Gifu Prefectural General Medical Center, 4-6-1 Noisshiki, Gifu-city, Gifu-prefecture 500-8717, Japan; Department of Surgery, Gifu Prefectural General Medical Center, 4-6-1 Noisshiki, Gifu-city, Gifu-prefecture 500-8717, Japan; Department of Surgery, Gifu Prefectural General Medical Center, 4-6-1 Noisshiki, Gifu-city, Gifu-prefecture 500-8717, Japan; Department of Surgery, Gifu Prefectural General Medical Center, 4-6-1 Noisshiki, Gifu-city, Gifu-prefecture 500-8717, Japan; Department of Surgery, Gifu Prefectural General Medical Center, 4-6-1 Noisshiki, Gifu-city, Gifu-prefecture 500-8717, Japan; Department of Surgery, Gifu Prefectural General Medical Center, 4-6-1 Noisshiki, Gifu-city, Gifu-prefecture 500-8717, Japan; Department of Surgery, Gifu Prefectural General Medical Center, 4-6-1 Noisshiki, Gifu-city, Gifu-prefecture 500-8717, Japan; Department of Surgery, Gifu Prefectural General Medical Center, 4-6-1 Noisshiki, Gifu-city, Gifu-prefecture 500-8717, Japan; Department of Surgery, Gifu Prefectural General Medical Center, 4-6-1 Noisshiki, Gifu-city, Gifu-prefecture 500-8717, Japan

**Keywords:** Zolbetuximab, conversion surgery, robotic-assisted surgery, CLDN18.2-positive gastric cancer

## Abstract

Zolbetuximab, a monoclonal antibody targeting Claudin (CLDN)18.2, has demonstrated clinical benefit in advanced gastric cancer, although reports of conversion surgery remain limited. A 59-year-old man with CLDN18.2-positive stage IV gastric cancer and suspected peritoneal dissemination received zolbetuximab with capecitabine and oxaliplatin. After six courses, imaging showed marked tumor regression. Staging laparoscopy revealed no peritoneal metastasis, and laparoscopic distal gastrectomy achieved R0 resection. Histopathology demonstrated a grade 2b response, and the final stage was ypStage IB. The patient remains recurrence-free at 12 months. Although conversion surgery was feasible, significant intraoperative bleeding occurred, likely due to treatment-related fibrosis and tissue fragility. This case suggests that zolbetuximab-based chemotherapy may enable conversion surgery in selected patients. Careful perioperative management is essential, and further studies are needed.

## Introduction

Gastric cancer remains a major global health burden and is frequently diagnosed at an advanced stage. The prognosis of initially unresectable stage IV disease remains poor despite advances in systemic therapy, with reported 5-year survival rates of approximately 7% [1]. In recent years, treatment options have expanded with the introduction of biomarker-driven targeted therapies and immune checkpoint inhibitors, improving clinical outcomes [2].

Conversion surgery, defined as curative resection after a favorable response to systemic therapy, has emerged as a promising treatment strategy in selected patients [3]. However, reports following CLDN18.2-targeted therapy remain limited. We report a case of CLDN18.2-positive stage IV gastric cancer with suspected peritoneal dissemination that responded to zolbetuximab plus CapeOX (capecitabine and oxaliplatin) therapy, enabling successful R0 resection.

## Case report

A 59-year-old man was referred to our hospital after an abnormal finding on an upper gastrointestinal series. Upper endoscopy revealed an ulcerative lesion in the greater curvature of the lower gastric body (Fig. 1), and biopsy showed poorly differentiated adenocarcinoma. Laboratory tests demonstrated anemia (hemoglobin 9.6 g/dl), elevated carbohydrate antigen 19–9, and normal carcinoembryonic antigen.

**Figure 1:**
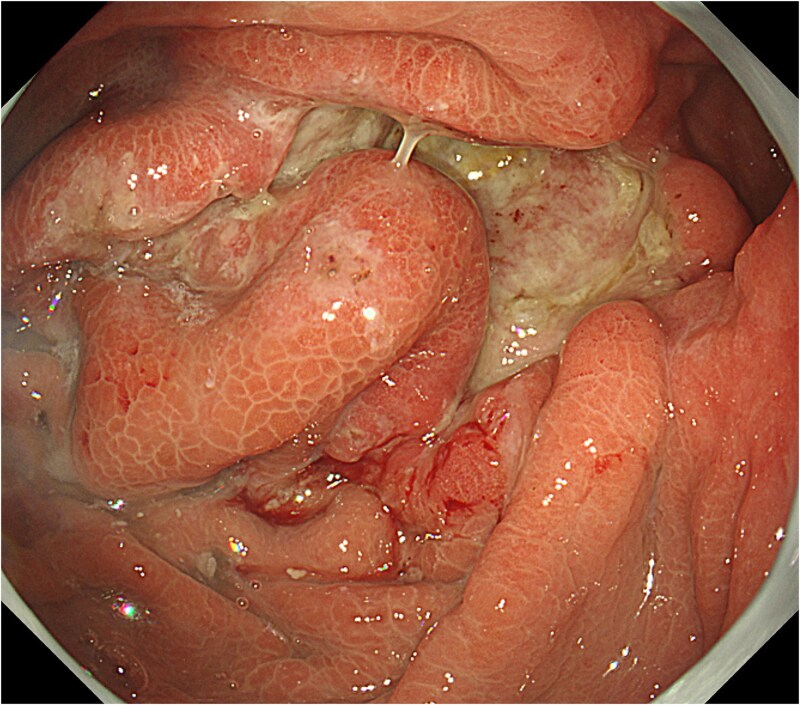
Upper gastrointestinal endoscopy showing an ulcerative type 3 lesion on the greater curvature of the lower gastric body.

Contrast-enhanced computed tomography (CECT) showed gastric wall thickening with multiple enlarged lymph nodes and omental nodules suggestive of peritoneal dissemination (Fig. 2a and b). A metastatic lymph node demonstrated invasion into the pancreatic head, with partial infiltration into the gastrocolic trunk (GCT). The clinical diagnosis was cT4b cN+ cM1(P1) cStage IVB. Immunohistochemistry demonstrated HER2 negativity and CLDN18.2 positivity.

**Figure 2:**
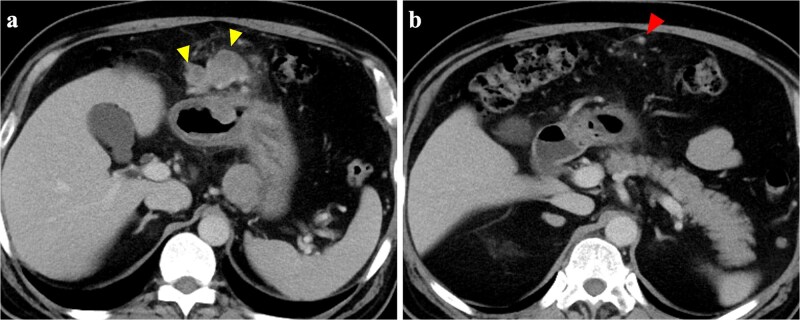
CECT at diagnosis. (a) Gastric wall thickening with regional lymph node enlargement. (b) Omental nodules suggestive of peritoneal dissemination.

The tumor was considered unresectable, and zolbetuximab combined with CapeOX was initiated. After five cycles, tumor markers normalized, and CECT showed marked tumor regression with disappearance of the suspected peritoneal lesions (Fig. 3a and b). Follow-up endoscopy revealed ulcer healing and re-epithelialization (Fig. 3c). Staging laparoscopy confirmed no peritoneal dissemination (Fig. 3d–f).

**Figure 3:**
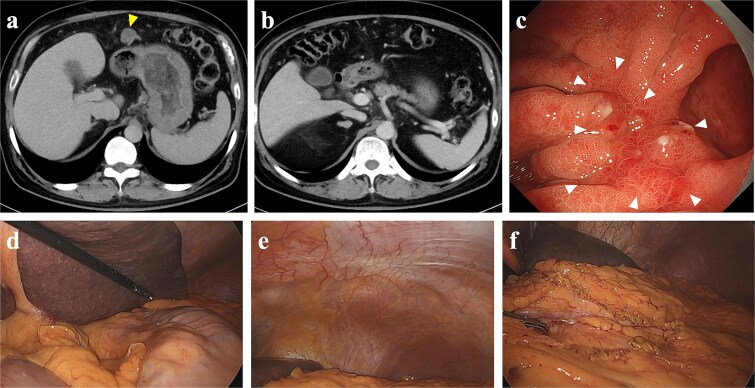
Post-treatment assessment. (a) CT showing tumor regression and lymph node reduction. (b) Disappearance of omental nodules. (c) Endoscopy showing ulcer healing and tumor regression. (d) Laparoscopy showing chemotherapy-related serosal changes. (e, f) No peritoneal dissemination.

Laparoscopic distal gastrectomy with D2 lymphadenectomy was performed. Fibrotic lymph nodes adherent to the GCT complicated dissection (Fig. 4) and resulted in substantial intraoperative bleeding (2700 ml).

**Figure 4:**
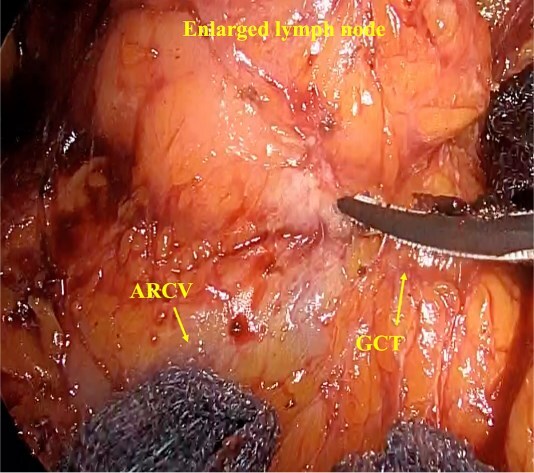
Intraoperative findings demonstrating an enlarged lymph node adherent to the GCT, with suspected invasion. ARCV: Accessory right colic vein.

Histopathological examination showed marked tumor regression with minimal residual poorly differentiated adenocarcinoma (Fig. 5). The pathological stage was ypT1b ypN1 ypM0 (ypStage IB). The postoperative course was uneventful, and the patient remains recurrence-free at 12 months.

**Figure 5:**
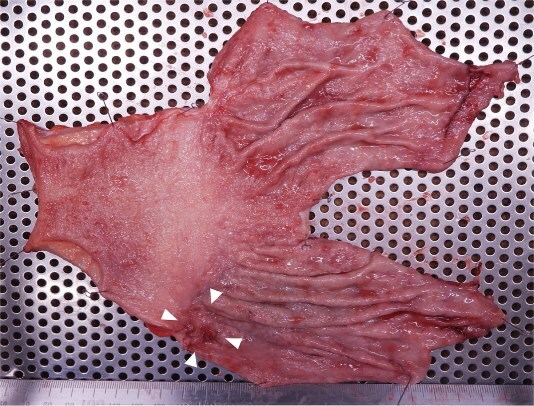
Pathological findings. Treatment-related scarring of the primary lesion. Residual poorly differentiated adenocarcinoma (grade 2b response).

## Discussion

CLDN18.2 is a tight junction protein expressed in gastric epithelial cells and has emerged as a promising therapeutic target in gastric cancer [4]. Disruption of epithelial polarity in malignant cells leads to exposure of CLDN18.2 on the tumor surface, enabling targeted therapy. Zolbetuximab, an anti-CLDN18.2 monoclonal antibody, exerts antitumor effects through antibody-dependent cellular cytotoxicity and complement-dependent cytotoxicity. Phase III trials have demonstrated improved clinical outcomes when zolbetuximab is combined with chemotherapy [5, 6], suggesting its potential role in tumor downstaging and facilitating conversion surgery. However, reports of conversion surgery after CLDN18.2-targeted therapy remain limited [7–9].

Conversion surgery has gained attention as a treatment option for selected patients with initially unresectable stage IV gastric cancer who respond to systemic therapy. Retrospective studies have suggested improved survival in patients who achieve R0 resection after chemotherapy. In the present case, the tumor was initially considered unresectable due to suspected pancreatic invasion, extensive lymph node metastases, and possible peritoneal dissemination. Following zolbetuximab-based chemotherapy, marked tumor regression was achieved, allowing successful R0 resection. This case highlights the potential role of CLDN18.2-targeted therapy as part of a multimodal treatment strategy aimed at curative resection.

From a perioperative perspective, several important considerations should be emphasized. First, gastrointestinal toxicity and early-onset gastritis associated with zolbetuximab may lead to anorexia and hypoalbuminemia. Impaired nutritional status is a well-established risk factor for postoperative complications such as surgical site infection, anastomotic leakage, and pneumonia [10, 11]. In the present case, hypoalbuminemia was observed and may have been related to protein loss secondary to gastritis. Histopathological findings suggested mucosal injury and regeneration. Although nutritional support was not required, careful monitoring of nutritional status is essential, and timely nutritional intervention should be considered when oral intake is insufficient.

Second, chemotherapy-induced tissue changes may increase surgical difficulty. In this case, fibrotic lymph nodes adherent to the GCT caused significant intraoperative bleeding and difficulty in achieving hemostasis. Tumor regression following systemic therapy may induce inflammatory reactions and fibrotic changes in surrounding tissues, obscuring anatomical planes and complicating dissection. In addition, therapy-associated hypoalbuminemia may contribute to tissue edema and fragility. Intraoperatively, edematous tissue made hemostasis difficult even with an ultrasonic device. These factors likely contributed to the substantial blood loss observed in this case.

These findings underscore an important limitation of conversion surgery. While tumor shrinkage may facilitate resectability, treatment-related tissue alterations may paradoxically increase operative complexity and risk. Surgeons should anticipate difficult dissection and potential bleeding, particularly in cases involving major vascular structures. Careful intraoperative judgment is required, and conversion to open surgery should not be hesitated when necessary to ensure patient safety.

Furthermore, the efficacy of zolbetuximab appears to be independent of programmed death-ligand 1 expression, suggesting that CLDN18.2 may serve as an independent predictive biomarker [12]. Even in cases where microsatellite instability or combined positive score cannot be assessed due to limited tissue availability, CLDN18.2 expression may provide a rational basis for treatment selection. Taken together, this case suggests that zolbetuximab-based chemotherapy can enable conversion surgery in selected patients with advanced gastric cancer. However, careful patient selection, meticulous perioperative management, and awareness of increased surgical difficulty are essential to optimize outcomes. Further accumulation of cases is required to clarify the role of conversion surgery following CLDN18.2-targeted therapy.
